# Temporal and seasonal trends in public interest in nonalcoholic beverages and lifestyles: A Google Trends analysis, 2004–2026

**DOI:** 10.1371/journal.pone.0361032

**Published:** 2026-10-29

**Authors:** Ying Tian, Dylan K. Richards, Melissa J. Cox

**Affiliations:** 1 Department of Health Behavior, Gillings School of Global Public Health, University of North Carolina at Chapel Hill, Chapel Hill, North Carolina, United States of America; 2 Department of Psychology, University of Georgia, Athens, Georgia, United States of America; University of South Carolina, UNITED STATES OF AMERICA

## Abstract

**Background:**

Growing awareness of alcohol harms and health consciousness has increased public interest in reducing alcohol consumption. However, little is known about how public interest in nonalcoholic beverages and lifestyles has changed over time. This study analyzed long-term and seasonal patterns in Google searches related to nonalcoholic beverages and lifestyles in the US from 2004 to 2026.

**Method:**

Google Trends data assessed monthly relative search volume (RSV) for five alcohol reduction–related search terms representing two categories: non-alcohol lifestyles (“quit drinking”, “Dry January”, and “sober curious”) and nonalcoholic beverages (“non alcoholic” and “mocktail”). Descriptive analyses were conducted to assess temporal patterns in RSV. Long-term trends, seasonal patterns, and changes in interest associated with COVID-19 were analyzed using quadratic regression and segmented regression models with Newey–West standard errors to address autocorrelation and heteroskedasticity.

**Results:**

Public interest in both alcohol reduction–related lifestyles and nonalcoholic beverages increased substantially over the study period. Beverage-related searches demonstrated more rapid growth following the COVID-19 pandemic, with significant positive post-pandemic trend changes observed for both “nonalcoholic” (β = 0.564, p < 0.001) and “mocktail” (β = 1.103, p < 0.001) terms, whereas lifestyle-related searches remained stable or grew slowly after the pandemic. Seasonal analyses showed lifestyle search “quit drinking” peaked in January, while beverage search “non alcoholic” peaked in December.

**Conclusions:**

Public interest in alcohol reduction via nonalcoholic beverages and lifestyles has risen, reflecting changing cultural and behavioral attitudes towards alcohol. These findings highlight the potential of Google search behavior to provide real-time insights into changing alcohol-related attitudes, harm reduction behaviors, and interest in no- and low-alcohol (NoLo) alternatives.

## 1 Introduction

Alcohol is the most widely used substance among the U.S. population aged 12 and older [1]. Excessive alcohol use is associated with a range of adverse health outcomes, including acute (e.g., motor vehicle crashes) and chronic conditions (e.g., cardiovascular disease, liver disease, seven types of cancers, and alcohol use disorder (AUD)) [2,3]. In 2021, excessive alcohol use accounted for approximately 178,000 deaths in the United States, or 488 deaths per day, making it a leading preventable cause of death [1].

Alcohol consumption is influenced by changing social, psychological, and environmental conditions [4,5]. The COVID-19 pandemic represented a major societal disruption that significantly altered alcohol consumption patterns in the United States. Alcohol use patterns shifted during the COVID-19 pandemic, with increases in drinking frequency and heavy drinking observed among women and individuals experiencing higher levels of stress and mental health challenges [6–9]. However, emerging evidence suggests that pandemic peaks in alcohol consumption are declining. The prevalence of adults reporting alcohol consumption declined from 62% in 2023 to 54% in 2025 [10,11]. Among these, reductions were more significant for women (11%), young adults (9%), and non-Hispanic White adults (11%) [10]. Drinking intensity also decreased, with the average past-week consumption dropping to 2.8 drinks (down from 4.0 in 2023) [10]. More people reported abstaining entirely, with 40% indicating not consuming alcohol in the past week, the highest proportion since 2000 [10]. Furthermore, by 2025, approximately 65% of U.S. adults reported intentions to reduce their alcohol consumption [12]. Regression from pandemic-related peaks in alcohol consumption, coupled with broader societal shifts in attitudes toward alcohol, may have created a unique context that fostered widespread interest in drinking less.

These behavioral shifts may reflect accumulating evidence that has challenged the long-standing belief that moderate alcohol consumption is beneficial for health. Current studies suggest that no level of alcohol consumption minimizes overall health risk (or protective) and that previously observed protective effects may largely reflect methodological biases [13,14]. In the US, perceived harm of moderate alcohol consumption (≤1 drink per day for females, ≤ 2 for males) increased from 25% in 2001 to 53% in 2025, with greater increases among young adults (31% to 66%), and higher perceived harm among women than men (60% vs. 47%) [10]. These changing perceptions are not limited to adults. Research also suggests that adolescents aged 14–17 are increasingly recognizing the negative consequences of any alcohol use, including health risks, injury, and reduced productivity or functioning [15].

Beyond increasing awareness of alcohol-related health risks, recent years have also seen the emergence of sobriety-oriented cultural trends and temporary abstinence campaigns [16,17]. The “Sober Curious” movement promotes a more mindful and health-conscious approach to drinking, and campaigns such as “Dry January” and “Sober October” encourage individuals to abstain from alcohol for a defined period [16,18]. These campaigns have gained substantial public engagement, with Dry January attracting millions of participants worldwide each year and showing a 36% increase in participation in 2025 compared with the previous year [19,20]. Research further suggests that participation in temporary alcohol abstinence challenges is associated with subsequent reductions in alcohol use [16,18]. For example, among U.S. young adults aged 18–29 years, approximately half reported reduced alcohol consumption following participation in a temporary alcohol abstinence challenge, with nearly 15% reporting continued abstinence after the challenge period [16]. Together, these trends suggest a broader cultural shift toward more health-conscious and alcohol-reducing lifestyles.

These behavioral and perceptual shifts are accompanied by growing interest in alternatives to traditional alcohol consumption. In recent years, nonalcoholic beverages have emerged as a rapidly expanding market [21]. In 2023, the global nonalcoholic beverage market was valued at approximately $35 billion and is projected to reach $46 billion by 2027, indicating substantial and sustained growth [22]. In response, industry stakeholders have expanded the development and marketing of no- and low-alcohol (NoLo) beverages [22,23]. Key beverage categories include nonalcoholic beer, wine, and spirits, with nonalcoholic beer accounting for the largest share of sales, though all categories continue to grow [21,24–26].

Collectively, these recent shifts in alcohol consumption behaviors, perceptions of alcohol, and non-alcohol beverage sales indicate a changing alcohol environment. To more fully capture these shifts, real-time data are needed. The current study uses Google Trends data to explore trends in Google searches related to sober lifestyles and nonalcoholic beverages. Google Trends is an open-access platform that enables the analysis of temporal and geographic patterns in Google Search activity for selected terms [27]. It provides real-time, population-level data on health-related information-seeking behavior, helping detect emerging trends that traditional surveys might miss [28]. Understanding these trends can provide valuable insights into emerging health behaviors and inform public health policy and intervention efforts.

## 2 Method

### 2.1 Search term development

The research team categorized non-alcohol–related search terms into two groups: nonalcoholic beverages and nonalcoholic lifestyle. An initial list of terms was generated by the research team. For the beverage group, we included mocktail, nonalcoholic, alcohol-free, zero alcohol, low alcohol, and alcohol substitute drink. For the lifestyle group, we included Dry January, Dry July, Sober October, quit drinking, damp-drinking, and alcohol reduction.

All initial search terms were entered into Google Trends, and the platform’s “Related queries” feature was reviewed to identify additional candidate search terms based on users’ search behavior. Data were examined from January 1, 2004, to February 27, 2026, in the United States, encompassing the entire period available through Google Trends at the time of data collection. Thus, February 2026 represents a partial month, with data available through February 27. Searches related to nonalcoholic beverages were under the “nonalcoholic beverages” category, while those related to nonalcoholic lifestyle were under the “Health” category. This process generated additional related search terms, including nonalcoholic drink, nonalcoholic beverage, zero proof, alcohol free beer, alcohol free wine, and alcohol-free spirits in the beverage group and sober curious for the lifestyle group. Next, the research team manually reviewed Google Search results, news/media articles, and online discussions related to alcohol reduction and nonalcoholic beverages to identify commonly used terms and phrases not captured in the initial Google Trends search. This process identified eleven additional beverage-related terms which were then evaluated using Google Trends to identify the top related searches, which returned two additional terms.

Next, all candidate terms from Google Trends-related searches and manual identification were entered into Google Trends under the nonalcoholic beverages and health category to generate the relative search volume over the study period for each term within each of the two categories. Since Google Trends can download aggregate data for only up to 5 terms, each term was downloaded individually. The research team then consulted a health science librarian with expertise in Google Trends analysis, who reviewed the candidate terms and confirmed no additional relevant search terms were needed. Candidate search terms were evaluated based on conceptual relevance, sufficient search activity, semantic distinctiveness, and expert consultation. Terms with insufficient Google Trends data or substantial conceptual overlap with other candidate terms were excluded to minimize redundancy. Accordingly, “nonalcoholic” and “mocktail” were chosen for the nonalcoholic beverages category, and “quit drinking”, “sober curious” and “Dry January” were selected for the nonalcoholic lifestyle category. The complete search strategy is provided in the S1 and S2 Tables.

### 2.2 Data extraction

Search interest was measured using relative search volume (RSV) obtained from Google Trends. RSV represents the share of searches for a specific term relative to all searches within the selected region and time and is normalized on a 0–100 scale, where 100 indicates the point of greatest relative search popularity. We downloaded the monthly RSV for each of the five terms every day from February 21, 2026, to February 27, 2026. For each search term, data were downloaded independently once per day at the same time over a seven-day period. Consistent with previous Google Trends studies, the monthly RSV used in the analysis was calculated as the average of these seven downloads to reduce sampling-related variability in the reported values [9,29–31]. This study used publicly accessible, anonymized data and therefore did not require institutional ethical approval.

### 2.3 Data analysis

Monthly RSV data were analyzed to examine temporal patterns in search interest for the five terms. Descriptive analyses were first conducted to visualize trends over time. Line plots were generated to display monthly RSV trajectories for each search term across the study period. Annual mean RSV values for each term were calculated to summarize year-to-year variation in search interest.

To quantify long-term trends, RSV was regressed on continuous time measured in years from January 2004, with monthly observations represented as fractional years. Linear and quadratic specifications were compared, with calendar-month indicators included to account for seasonality. Quadratic terms were retained when statistically significant and when the quadratic specification improved model fit. Model diagnostics included residual inspection, Ramsey RESET tests, and tests for autocorrelation (ACF/PACF plots, Breusch–Godfrey, and Durbin–Watson tests). Final models used Newey–West standard errors with a 12-month lag to account for heteroskedasticity and serial correlation. As a sensitivity analysis, restricted cubic spline models with four knots at the 5th, 35th, 65th, and 95th percentiles of the time distribution were estimated using the same time scale, calendar-month adjustment, and Newey–West standard errors.

Similarly, seasonal patterns were assessed by estimating regression models for each search term with RSV regressed on calendar year (including linear and quadratic terms), as well as month-of-year indicators, using Newey–West standard errors (lag = 12). Overall seasonality was evaluated using a joint Wald test of the month-of-year indicators to determine whether adjusted RSV differed systematically across months after accounting for long-term trends. Adjusted monthly RSV estimates were then obtained using marginal predictions and visualized with margins of plots.

To examine the changes of the COVID-19 pandemic (March 2020), we used segmented regression models that included a continuous monthly time variable, an indicator for the post-COVID period, and an interaction term representing post-COVID trend changes. Monthly indicators were included to control for seasonality. Newey–West standard errors (lag = 12) were applied to account for autocorrelation. In addition, restricted cubic spline models were estimated to assess the robustness of the long-term temporal patterns to a more flexible specification of time.

## 3 Results

### 3.1 Descriptive trends

Monthly RSV trends for the five non-alcohol–related search terms are presented in Fig 1. “Quit drinking” consistently showed the highest search interest among lifestyle-related terms, whereas “Dry January” and “sober curious” increased primarily in recent years. For beverage-related terms, both “nonalcoholic” and “mocktail” demonstrated clear upward trends over the study period. Annual mean RSV estimates for each search term are presented in S3 Table.

**Fig 1:**
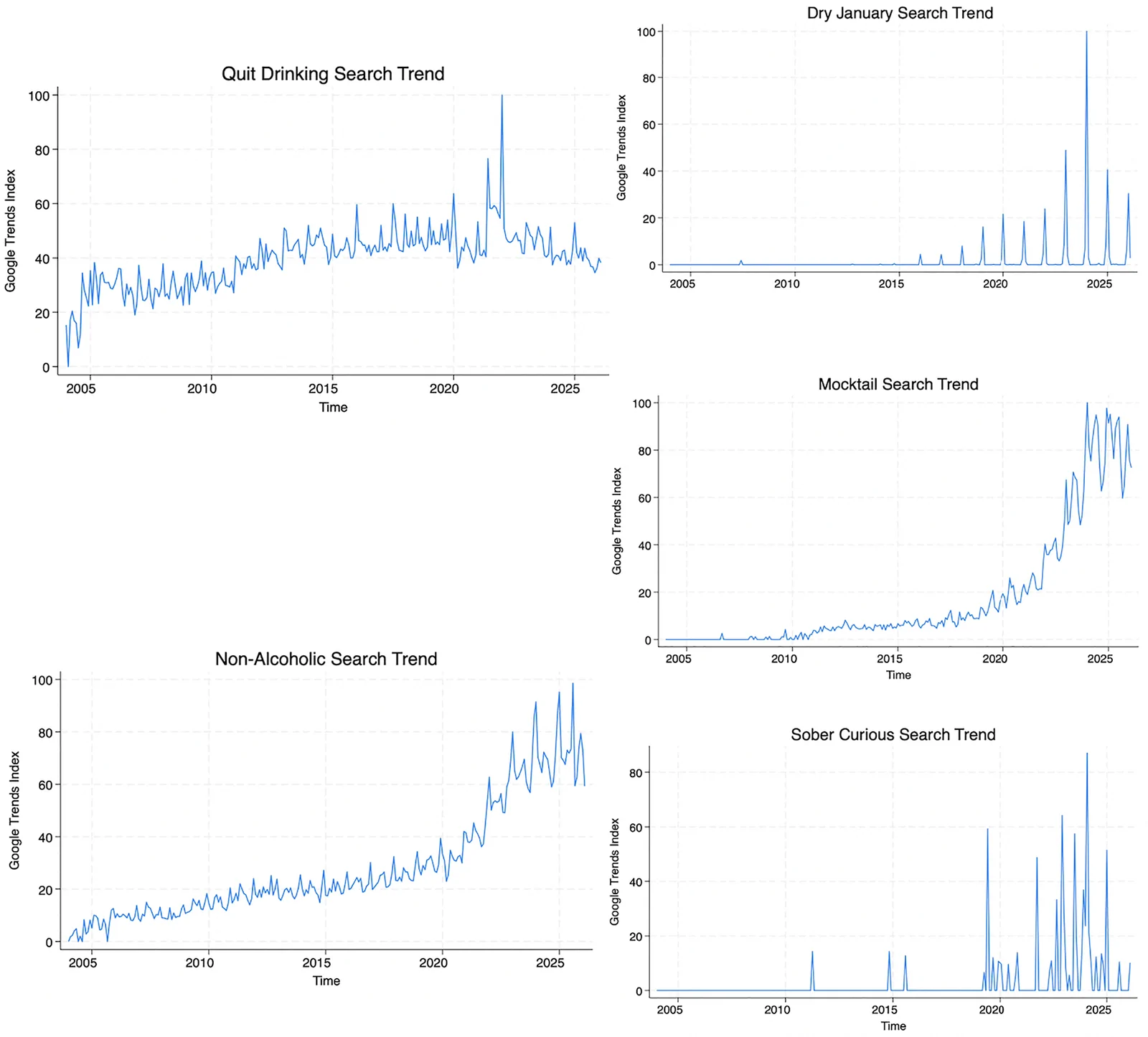
Descriptive trends in non-alcohol-related beverage and lifestyle searches. *Notes. Monthly data were obtained from Google Trends in the United States from January 2004 to February 2026. Google Trends indices are normalized from 0 to 100, representing relative search interest over time for each term.

### 3.2 Regression analysis of long-term trends in annual RSV

Table 1 presents results from regression models with Newey–West standard errors to assess long-term temporal patterns in search interest. Across all search terms, evidence of non-linear trends was observed.

**Table 1:** Regression analyses of long-term trends in monthly RSV for alcohol reduction–related search terms.

Search term	Linear term β (95% CI)	Quadratic term β^2^ (95% CI)	Overall model F statistic	Trend pattern
“quit drinking”	3.654 (2,646 4.663)	− 0.115 (− 0.156, − 0.075)	F (13, 252) = 17.39, p < 0.001	concave
“sober curious”	−0.453 (−1.290, 0.384)	0.044 (−0.005, 0.093)	F (13, 252) = 3.16, p < 0.05	No statistically supported curvature
“dry January”	−0.318 (−0.590, −0.045)	0.027 (0.010, 0.043)	F (13, 252) = 4.81, p < 0.001	convex
“non alcoholic”	−1.391 (−2.666, −0.117)	0.196 (0.140, 0.251)	F (13, 252) = 67.15, p < 0.001	convex
“mocktail”	−4.619 (−6.533, −2.704)	0.354 (0.262, 0.447)	F (13, 252) = 16.64, p < 0.001	convex

*Note. RSV = relative search volume; CI = confidence interval. Models were estimated using monthly observations over the full observation period (January 2004–February 2026; N = 266). Time was modeled as a continuous variable measured in years from January 2004, with monthly observations represented as fractional years. All models included calendar-month indicators to account for seasonality and were estimated using Newey–West heteroskedasticity- and autocorrelation-consistent standard errors with a 12-month lag. Positive quadratic coefficients indicate convex temporal patterns, whereas negative quadratic coefficients indicate concave temporal patterns. All values were rounded to three decimal places.

For lifestyle-related search terms, “quit drinking” exhibited a concave temporal pattern (β = 3.654, 95% CI: 2.646 to 4.663; β^2^ = −0.115, 95% CI: −0.156 to −0.075; F (13, 252) = 17.39, p < 0.001), indicating search interest grew over time, but the rate slowed in later years. “Sober curious” showed a significant overall time effect (F (13, 252) = 3.16, p < 0.05), but neither the linear nor quadratic term was statistically significant. Although the quadratic coefficient was positive, the evidence was insufficient to characterize the trend as definitively convex. “Dry January” exhibited a convex pattern (β = −0.318, 95% CI: −0.590 to −0.045; β^2^ = 0.027, 95% CI: 0.010 to 0.043; F (13, 252) = 4.81, p < 0.001), indicating accelerating increases over time.

For beverage-related search terms, both “nonalcoholic” (β = −1.391, 95% CI: −2.666 to −0.117; β^2^ = 0.196, 95% CI: 0.140 to 0.251; F (13, 252) = 67.15, p < 0.001) and “mocktail” (β = −4.619, 95% CI: −6.533 to −2.704; β^2^ = 0.354, 95% CI: 0.262 to 0.447; F (13, 252) = 16.64, p < 0.001) exhibited strong convex patterns, indicating rapidly accelerating increases in search interest over time.

Restricted cubic spline sensitivity analyses yielded temporal patterns broadly consistent with the primary models, supporting the robustness of the findings to a more flexible specification of time (S5 Fig).

### 3.3 Seasonal patterns in monthly RSV across search terms

As indicated in Fig 2, seasonal patterns in search interest were examined using regression models including month indicators with Newey–West standard errors to account for autocorrelation. A significant seasonal pattern was observed for “quit drinking” searches (F (11, 252) = 8.70, p < 0.001). Adjusted monthly estimates indicated that search interest peaked in January (47.789, 95% CI: 43.278 to 52.299). Searches for nonalcoholic beverages also exhibited a significant seasonal pattern (F (11, 252) = 10.52, p < 0.001), with adjusted estimates indicating a peak in December (35.834, 95% CI: 32.791 to 38.877). However, no significant seasonal patterns were observed for “mocktail” (*p* = .251), “sober curious” (*p* = .478), or “Dry January” (*p* = .498). Detailed results of the seasonal analyses are provided in S4 Table.

**Fig 2:**
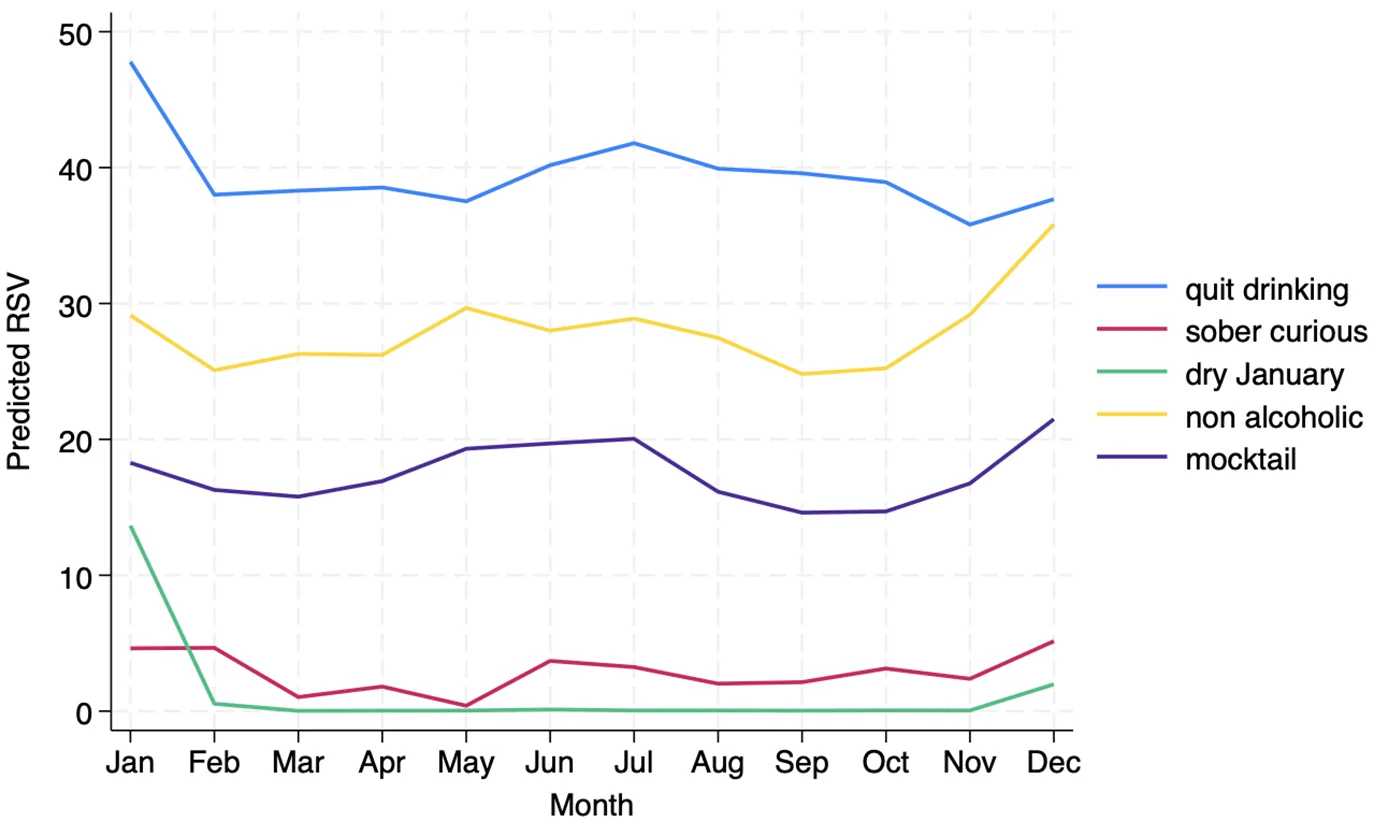
Seasonal patterns of non-alcohol-related searches. *Notes. Lines represent adjusted monthly relative search volume (RSV) estimates by calendar month using U.S. Google Trends data from January 2004 through February 2026. Models adjusted for long-term temporal trends and used Newey–West standard errors with a 12-month lag.

### 3.4 Pre- and post-COVID-19 trends in annual RSV

Results of segmented regression analyses are presented in Table 2. Search interest in “quit drinking” showed a significant increasing trend prior to the pandemic (β = 0.152, 95% CI: 0.122 to 0.182, p < 0.001). At the onset of COVID-19, no significant immediate change in search interest was observed (β = −1.027, 95% CI: −11.410 to 9.355, p = 0.846). However, the post-pandemic trend showed a significant decrease (β = −0.306, 95% CI: −0.500 to −0.111, p = 0.002). Search interest in “Dry January” showed a significant increasing trend prior to the COVID-19 pandemic (β = 0.008, 95% CI: 0.002–0.014, p = 0.005). There was no evidence of an immediate change in search interest at the onset of the pandemic (β = 0.807, 95% CI: −1.157 to 2.771, p = 0.419). The post-pandemic trend showed a marginal but non-significant increase compared to the pre-pandemic trend (β = 0.064, 95% CI: −0.003 to 0.131, p = 0.062). For “sober curious,” neither the pre-pandemic trend nor the immediate level change and post-pandemic slope change were statistically significant (all p > 0.05).

**Table 2:** Segmented regression analyses of pre- and post-COVID-19 trends in monthly RSV.

Search term	Pre-pandemic trend β (95% CI)	Immediate level change β (95% CI)	Post-pandemic trend change β (95% CI)	Overall model F statistic
Quit drinking	0.152 (0.122, 0.182)	−1.027 (−11.410, 9.355)	−0.306 (−0.500, −0.111)	F (14, 251) = 38.73, p < 0.001
Dry January	0.008 (0.002, 0.014)	0.807 (−1.157, 2.771)	0.064 (−0.003, 0.131)	F (14, 251) = 4.73, p < 0.001
Sober curious	0.017 (−0.004, 0.038)	3.258 (−4.382, 10.899)	0.064 (−0.151, 0.278)	F (14, 251) = 3.37, p < 0.001
Non-alcoholic	0.125 (0.113, 0.138)	4.185 (−2.397, 10.769)	0.564 (0.397, 0.731)	F (14, 251) = 209.57, p < 0.001
Mocktail	0.073 (0.055, 0.090)	−0.384 (−7.471, 6.702)	1.103 (0.854, 1.352)	F (14, 251) = 56.15, p < 0.001

*Note. Segmented regression models were estimated using monthly observations from January 2004 through February 2026, with March 2020 specified as the pandemic-related breakpoint. Models included month-of-year indicators to account for seasonality and used Newey–West heteroskedasticity- and autocorrelation-consistent standard errors with a 12-month lag. The pre-pandemic trend represents the estimated monthly trend before March 2020, the immediate level change represents the estimated change in RSV at the breakpoint, and the post-pandemic trend change represents the estimated change in slope following the breakpoint relative to the pre-pandemic trend. RSV = relative search volume; CI = confidence interval.

Among nonalcoholic beverage-related terms, search interest in “nonalcoholic” beverages showed a significant increasing trend prior to the COVID-19 pandemic (β = 0.125, 95% CI: 0.113 to 0.138, p < 0.001). There was no evidence of an immediate change in search interest at the onset of the pandemic (β = 4.185, 95% CI: −2.397 to 10.769, p = 0.212). However, the post-pandemic trend increased significantly compared to the pre-pandemic trend (β = 0.564, 95% CI: 0.397 to 0.731, p < 0.001).

Similarly, search interest in “mocktail” showed a significant increasing trend prior to the COVID-19 pandemic (β = 0.073, 95% CI: 0.055 to 0.090, p < 0.001). There was no evidence of an immediate change in search interest at the onset of the pandemic (β = −0.384, 95% CI: −7.471 to 6.702, p = 0.915). However, the post-pandemic trend increased significantly compared to the pre-pandemic trend (β = 1.103, 95% CI: 0.854 to 1.352, p < 0.001). All segmented regression trends comparing pre- and post- COVID are presented in Fig 3.

**Fig 3:**
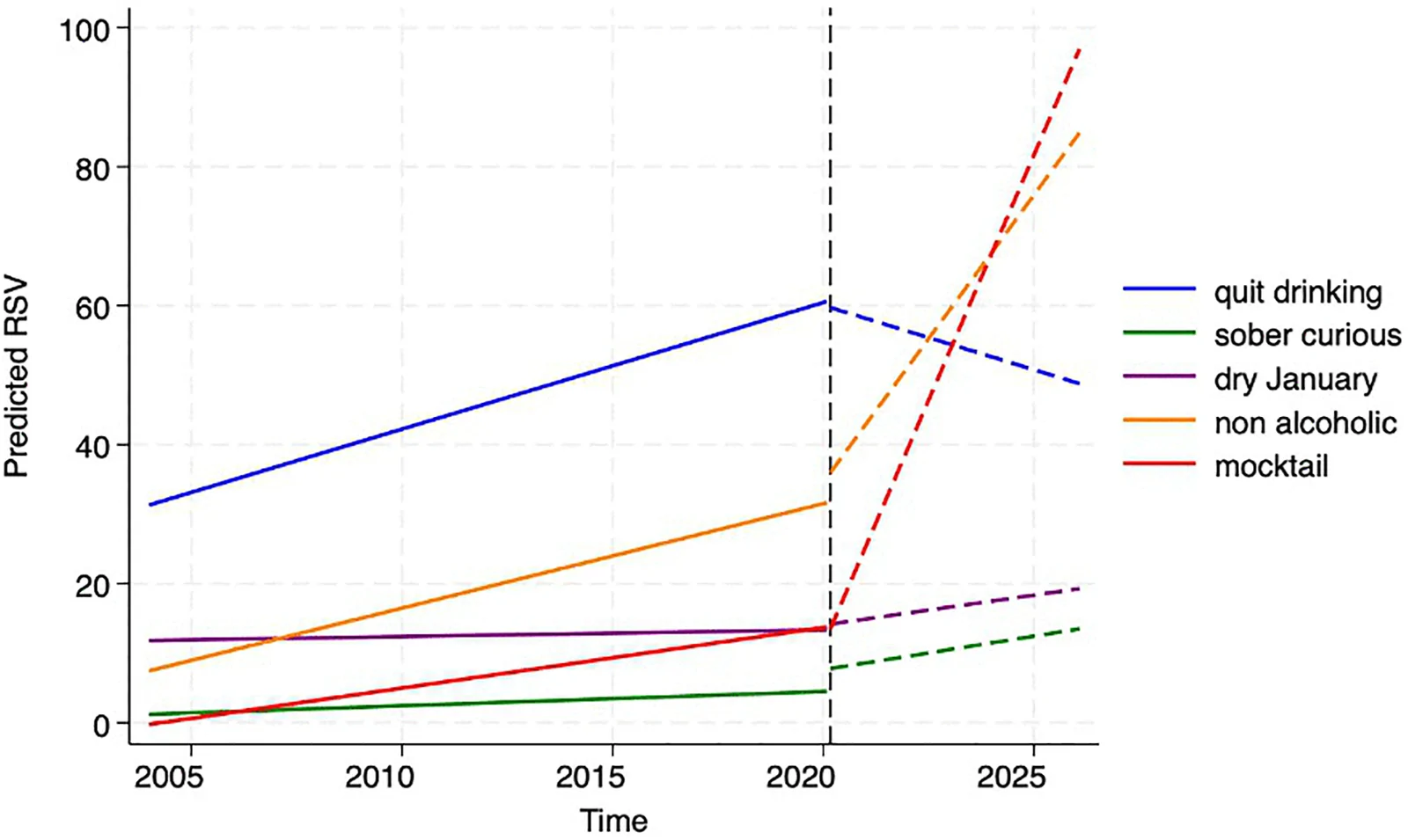
Interrupted time series trends in non-alcohol-related search interest before and after the onset of COVID-19. *Note. Lines represent monthly predicted relative search volume (RSV) values estimated from interrupted time series regression models using Google Trends data from January 2004 to February 2026 in the United States. The vertical dashed line indicates March 2020, marking the onset of the COVID-19 pandemic period. RSV values range from 0 to 100, with higher values indicating greater relative search interest.

Sensitivity analyses using restricted cubic splines showed temporal patterns largely aligning with the primary models, indicating that the findings remain robust even with a more flexible time specification (S6 Fig).

## 4 Discussion

This study examined long-term trends and seasonal patterns in public search interest in nonalcoholic beverages and lifestyles. Overall, the results indicate growing public interest in alcohol reduction–related searches, consistent with prior evidence of recent declines in alcohol use [9,10] and aligns with the expansion of the non-alcohol beverage market [18,19]. This trend is likely associated with multiple factors. First, accumulating evidence challenging the notion of a safe level of alcohol consumption may have been associated with greater public awareness of alcohol-related harms and changing perceptions regarding the health consequences of drinking. Further, growing public health efforts have prioritized communicating the health risks associated with alcohol consumption, including the increased risk of cancer even at low levels of use, as evidenced by the U.S. Surgeon General’s 2025 Advisory [32]. These shifts in awareness and perceptions may, in turn, have been associated with changes in drinking behaviors and increased interest in nonalcoholic alternatives. More than 80 percent of people prefer healthier lifestyles, with increasing demand for beverages that offer functional benefits, natural ingredients, and lower sugar levels [33–35]. Furthermore, the emergence of popular cultural movements, such as the “Dry January” movement, may have been associated with normalized reduced alcohol consumption and increased both curiosity about and acceptance of NoLo beverages [33–35].

Beverage-related searches increased more rapidly than lifestyle-oriented searches, particularly following the onset of the COVID-19 pandemic. While beverage-related terms exhibited accelerated growth, lifestyle-related searches remained relatively stable, with “quit drinking” even showing a deceleration in growth. On one hand, this divergence may partly associated with the differences in the populations represented by these search terms. Beverage-related terms may be related to interest from a broader consumer population, including individuals seeking healthier alternatives as well as those reducing alcohol consumption [36], whereas lifestyle-oriented terms such as “quit drinking,” “Dry January,” and “sober curious” may be more directly related to intentions to change drinking behavior [37]. Consequently, increasing interest in no- and low-alcohol beverages may be associated with broader consumer acceptance and adoption, whereas lifestyle-oriented searches may be more specifically related with motivation for alcohol behavior change [38].

On the other hand, this divergence may also be related to the differences in how individuals modify their drinking behaviors. Instead of complete abstinence, many may prefer more moderate approaches, including incorporating NoLo beverages into their daily lives. These choices may be associated with not only by health concerns but also by practical motivations, such as remaining able to drive safely, avoiding intoxication, and maintaining normal daily activities [26,39,40]. Some individuals may also alternate alcoholic and nonalcoholic beverages during drinking occasions, a practice that may be associated with lower acute alcohol-related harms while allowing continued participation in social drinking contexts. In this way, NoLo beverages may be related with both harm reduction and continued social engagement [26,39,41].

What’s more, this pattern is also consistent with core principles of behavioral economics, which suggest that individuals tend to favor behavioral options that require lower effort and entail lower perceived costs [42,43]. Compared with complete abstinence, choosing NoLo represents a lower-effort pathway for behavior change and may therefore be related to readily adopted [35,40]. In parallel, the rapid growth of the nonalcoholic beverage market has been associated with increased availability and accessibility of these beverages in everyday environments, factors that may be related to fewer perceived barriers to choosing nonalcoholic alternatives [44–47]. Experimental evidence suggests that exposure to nonalcoholic beverage advertising increase the likelihood of selecting these beverages [48]. Moreover, features of the retail environment, such as the relative proportion of nonalcoholic to alcoholic beverages on display, have been associated with consumer decision-making [41], with higher availability of nonalcoholic options associated with reduced selection of alcoholic beverages [49].

The observed divergence may also be related to the broader social and psychological impacts of the pandemic. Even in the context of growing health awareness, increased stress, reduced social support, and disruption to daily routines during the pandemic may have been associated with greater difficulty maintaining alcohol abstinence, particularly for individuals who use alcohol as a coping strategy [50–52]. Stress has been associated with lower self-regulatory capacity and greater preference for more immediate and less effortful behavioral options [53,54]. Under such conditions, individuals may be less likely to adopt more restrictive lifestyle changes, such as quitting alcohol entirely, as the benefits of abstinence are typically delayed and realized over the long term. In contrast, NoLo alcohol alternatives retain key aspects of the drinking experience, including sensory qualities and associated drinking rituals, and may therefore represent a more immediately reinforcing, attractive, and feasible option [39,40].

In addition to long-term trends, searches for “quit drinking” peaked at the beginning of the year, suggesting a seasonal pattern in health-related information seeking. This finding is consistent with prior studies that have found increases in Google searches for diets and visits to online smoking cessation programs [55,56]. Such patterns may be related to a “fresh start” effect associated with temporal landmarks, which can motivate individuals to reconsider past behaviors and initiate new health-related goals [57,58]. These moments are often associated with psychological separation from one’s past self, greater big-picture thinking, renewed motivation, and a greater sense of self-regulatory capacity, which can facilitate behavior change [59,60], potentially increasing interest in reducing or abstaining from alcohol [61–64]. In contrast, no significant seasonal patterns were observed for “sober curious” or “Dry January,” suggesting that seasonal variation was not consistently detectable for these terms across the full observation period from 2004 to 2026.

Unlike lifestyle-oriented searches, the patterns for non-alcoholic beverages seem to vary. Searches for “mocktail” did not show a clear seasonal pattern, indicating relatively stable interest throughout the year. However, searches for “nonalcoholic” beverages tended to peak in December. One possible explanation is that, compared to the term “mocktail,” the term “nonalcoholic” represents a broader, category-level search that may reflect information-seeking and decision-making processes, whereas more specific queries may capture narrower, consumption-oriented interests.

In addition, the December peak may be associated with the increased demand for substitutes during periods of heightened alcohol consumption, potentially reflecting greater awareness of alcohol-related harms and interest in alternatives that can replace alcoholic beverages in social settings [65,66]. In contexts where drinking is normative, individuals who are attempting to reduce or abstain from alcohol may face social pressure or concerns about social exclusion [67]. NoLo beverages may represent a socially acceptable alternative that allows individuals to participate in drinking occasions without consuming alcohol, potentially facilitating social inclusion while consistent with their personal drinking goals.

This study robustly examined long-term patterns in public interest related to alcohol reduction and nonalcoholic alternatives and provides a novel perspective on the intersection between alcohol reduction culture and growing interest in NoLo beverages. However, several limitations should also be considered. First, Google Trends measures relative search interest rather than actual alcohol use or reduction behaviors, and zero RSV values may reflect very low search activity rather than an absence of interest. Accordingly, the observed search trends should not be interpreted as direct evidence of corresponding behavioral changes. Second, because the analyses were based on aggregated national-level data, the results may be subject to ecological fallacy and cannot be interpreted at the individual level. Third, search term selection may also introduce bias, and because longitudinal data for excluded candidate terms were not retained, sensitivity analyses using alternative term sets could not be conducted. Fourth, although restricted cubic spline analyses supported the robustness of the findings to a more flexible specification of time, no single model can fully capture complex temporal patterns. Finally, segmented regression characterized changes surrounding the onset of the COVID-19 pandemic rather than establishing a causal effect. Although alternative breakpoint analyses yielded consistent findings, concurrent societal, policy, media, and market changes could not be independently accounted for and may have contributed to the observed trends.

## 5 Conclusion

This study examined long-term temporal and seasonal patterns in Google searches related to alcohol reduction and nonalcoholic alternatives in the United States from 2004 to 2026. Findings suggest that public interest in both alcohol reduction–related lifestyles and nonalcoholic beverages increased substantially over the study period, with beverage-related searches demonstrating more rapid growth, particularly following the COVID-19 pandemic. Seasonal patterns were more term-specific, with searches for “quit drinking” peaking at the beginning of the year and searches for “nonalcoholic” peaking toward the end of the year. Overall, these findings suggest evolving cultural and behavioral approaches to alcohol reduction, including growing interest in more flexible and socially acceptable alternatives such as NoLo beverages. Insights from this study may contribute to broader understanding of alcohol reduction behaviors and inform future public health strategies, harm reduction efforts, and alcohol-related policy initiatives. Future studies should also investigate potential differences in alcohol reduction–related search behaviors across states and sociodemographic groups to better understand disparities in alcohol-related attitudes, behaviors, and access to nonalcoholic alternatives.

## Supporting information

S1 Table Candidate search terms and selection rationale. Candidate search terms considered for the non-alcoholic beverage and non-alcohol lifestyle categories, including relative search volume (RSV) and the rationale for retention or exclusion.
(DOCX)

S2 Table Google Trends search terms and categories. Final Google Trends search terms and corresponding themes, geographic region, search period, search categories, and search operators used in the analysis.
(DOCX)

S3 Table Annual mean RSV values for alcohol reduction-related search terms, 2004–2026. Annual mean monthly relative search volume (RSV) estimates for each retained search term in the United States from 2004 through 2026.
(DOCX)

S4 Table Seasonal patterns in monthly RSV across search terms. Results of seasonality analyses for the retained search terms, including overall seasonality tests and peak adjusted monthly RSV estimates for statistically significant seasonal patterns.
(DOCX)

S5 Fig Comparison of primary quadratic and restricted cubic spline models for long-term temporal trends. Comparison of long-term temporal patterns estimated using the primary quadratic models and restricted cubic spline sensitivity models for each retained search term.
(DOCX)

S6 Fig Long-term temporal trends estimated using restricted cubic spline and COVID-19 segmented regression models. Results of sensitivity analyses using restricted cubic spline and COVID-19 segmented regression models to assess the robustness of long-term temporal trends across the retained search terms.
(DOCX)
