# Supplementary material for: Temporal and seasonal trends in public interest in nonalcoholic beverages and lifestyles: A Google Trends analysis, 2004–2026
Source: PLoS One. 2026 Oct 29;21(10):e0361032. doi: 10.1371/journal.pone.0361032 (PMC13718958; doi:10.1371/journal.pone.0361032)
Supplement: Supplementary file 1 [file pone.0361032.s001.docx]

S1 Table. Candidate search terms and selection rationale

| Non-Alcoholic Beverages | RSV | Decision/rationale | Lifestyle | RSV | Decision/rationale |
| --- | --- | --- | --- | --- | --- |
| kombucha | 37.33834586 | Excluded - Limited specificity to alcohol reduction or substitution | **quit drinking** | **39.43984962** | **Retained - Representative term capturing drinking cessation** |
| **non alcoholic** | **29.10526316** | **Retained - broad representative term capturing general interest in nonalcoholic beverages** | alcohol reduction | 2.804511278 | Excluded - Broad conceptual overlap with selected alcohol-reduction lifestyle terms |
| alcohol free | 26.65789474 | Excluded – Product-specific term; broader beverage domain already represented | **sober curious** | **2.77443609** | **Retained - Conceptually distinct term representing a broader alcohol-reduction lifestyle orientation** |
| alcohol free wine | 24.19172932 | Excluded - semantic overlap with “nonalcoholic” | sober October | 2.255639098 | Excluded - Conceptual overlap with month-specific alcohol-abstinence campaigns; “Dry January” retained as the representative term based on its established use in the alcohol-abstinence literature |
| **mocktail** | **17.42481203** | **Retained - Conceptually distinct term representing alcohol-free mixed drinks** | temperance movement | 1.763157895 | Excluded - Refers primarily to a historical/social movement rather than contemporary alcohol-reduction behavior |
| alcohol free beer | 14.2180451 | Excluded - Product-specific term; broader beverage domain already represented | **Dry January** | **1.413533835** | **Retained - Representative and well-established month-specific alcohol-abstinence campaign** |
| zero proof | 8.962406015 | Excluded - Overlapping terminology; broader beverage domain already represented | alcohol-free lifestyle | 0.838345865 | Excluded - Conceptual overlap with selected broader alcohol-reduction lifestyle terms and sparse search activity |
| non alcoholic beverage | 5.54887218 | Excluded - Semantic overlap with “non alcoholic” | sober Spring | 0.545112782 | Excluded - Overlap with month-specific alcohol-abstinence campaigns and sparse search activity |
| zero alcohol | 3.879699248 | Excluded - Semantic overlap with “non alcoholic” | dry December | 0.518796992 | Excluded - Overlap with month-specific alcohol-abstinence campaigns and sparse search activity |
| non alcoholic drink | 2.909774436 | Excluded - Semantic overlap with “non alcoholic” | temperance bar | 0.511278195 | Excluded - Limited specificity to individual alcohol-reduction behavior or lifestyle and sparse search activity |
| low alcohol | 1.894736842 | Excluded - Represents reduced-alcohol rather than nonalcoholic products | alcohol free challenge | 0.37593985 | Excluded - Overlap with alcohol-abstinence challenge terminology and sparse activity |
| functional beverage | 0.443609023 | Excluded - Limited specificity to alcohol reduction or substitution and sparse search activity | damp-drinking | 0.37593985 | Excluded - Sparse search activity; broader alcohol-reduction construct already represented |
| NOLO drink | 0.398496241 | Excluded - Sparse search activity and overlap with broader selected terminology | damp-lifestyle | 0.37593985 | Excluded - Sparse search activity; broader alcohol-reduction construct already represented |
| alcohol substitute drinks | 0.37593985 | Excluded - Sparse search activity and broader beverage domain already represented | mindful drinking | 0.37593985 | Excluded - Sparse search activity; broader alcohol-reduction construct already represented |
| alternative beverage | 0.37593985 | Excluded - Limited specificity to nonalcoholic alcohol substitutes and sparse search activity | OYNB | 0.37593985 | Excluded - Sparse search activity and limited interpretability as a general alcohol-reduction search term |
| alcohol free spirit | N/A | Excluded - Insufficient Google Trends data | sober bar | 0.37593985 | Excluded - Sparse search activity and limited specificity to individual alcohol-reduction behavior |
|  |  |  | sobriety challenge | 0.37593985 | Excluded - Sparse search activity; overlaps with alcohol-abstinence challenge terminology |
|  |  |  | dry July | N/A | Excluded - Insufficient Google Trends data |

*Notes. RSV = the average monthly relative search volume; N/A = insufficient search data available from Google Trends. Google Trends data were collected for the United States from January 2004 through February 2026, with data retrieval conducted from February 21 to February 27, 2026.

Each term’s RSV was downloaded individually. RSV values were used to assess the availability and stability of search data rather than for direct comparisons of search volume across independently queried terms.

Candidate terms were evaluated based on conceptual relevance, sufficient search activity, semantic distinctiveness, and expert consultation. Because multiple search terms could represent the same underlying concept, conceptually overlapping terms were not simultaneously retained in order to minimize redundancy. When multiple candidate terms represented the same construct, representative terms were selected based on their conceptual fit, interpretability, search-data availability, and, where applicable, established use in the literature. Terms for which Google Trends did not provide sufficient data to generate a usable time series were excluded.
