# Supplementary material for: Temporal and seasonal trends in public interest in nonalcoholic beverages and lifestyles: A Google Trends analysis, 2004–2026
Source: PLoS One. 2026 Oct 29;21(10):e0361032. doi: 10.1371/journal.pone.0361032 (PMC13718958; doi:10.1371/journal.pone.0361032)
Supplement: Supplementary file 2 [file pone.0361032.s002.docx]

S2 Table. Google Trends search terms and categories

| Theme | Geographic Region | Search Time Period | Search Category | Search Term | Search Operators |
| --- | --- | --- | --- | --- | --- |
| Non-Alcohol Products | United States | 01/01/2004-02/27/2026 | Non-Alcoholic Beverages | “non alcoholic” | N/A |
|  |  |  |  | “mocktail” | N/A |
| Non-Alcohol Lifestyle | United States | 01/01/2004-02/27/2026 | Health | “quit drinking” | N/A |
|  |  |  |  | “sober curious” | N/A |
|  |  |  |  | “dry January” | N/A |

*Note. Search data were obtained from Google Trends for the United States between January 1, 2004 and February 27, 2026. Search operators were not applied to any search terms.
