# Supplementary material for: Temporal and seasonal trends in public interest in nonalcoholic beverages and lifestyles: A Google Trends analysis, 2004–2026
Source: PLoS One. 2026 Oct 29;21(10):e0361032. doi: 10.1371/journal.pone.0361032 (PMC13718958; doi:10.1371/journal.pone.0361032)
Supplement: Supplementary file 3 [file pone.0361032.s003.docx]

S3 Table. Annual mean RSV values for alcohol reduction-related search terms, 2004–2026

| Year | dry January | quit drinking | sober curious | mocktail | non alcoholic |
| --- | --- | --- | --- | --- | --- |
| 2004 | 0.000 | 17.881 | 0.000 | 0.000 | 3.190 |
| 2005 | 0.000 | 30.881 | 0.000 | 0.000 | 7.464 |
| 2006 | 0.000 | 28.190 | 0.000 | 0.214 | 9.821 |
| 2007 | 0.155 | 27.583 | 0.000 | 0.000 | 10.869 |
| 2008 | 0.000 | 29.000 | 0.000 | 0.488 | 10.179 |
| 2009 | 0.000 | 31.226 | 0.000 | 0.595 | 13.631 |
| 2010 | 0.000 | 30.952 | 0.000 | 1.266 | 14.881 |
| 2011 | 0.000 | 37.762 | 1.190 | 4.155 | 17.750 |
| 2012 | 0.0234 | 40.607 | 0.000 | 5.560 | 19.620 |
| 2013 | 0.000 | 44.071 | 0.000 | 4.905 | 18.870 |
| 2014 | 0.060 | 45.619 | 1.190 | 5.429 | 19.976 |
| 2015 | 0.000 | 42.976 | 1.060 | 6.833 | 20.523 |
| 2016 | 0.381 | 45.143 | 0.000 | 6.357 | 22.274 |
| 2017 | 0.357 | 46.548 | 0.000 | 8.226 | 23.679 |
| 2018 | 0.905 | 46.929 | 0.000 | 9.714 | 25.750 |
| 2019 | 1.560 | 48.131 | 7.381 | 14.286 | 29.750 |
| 2020 | 2.048 | 44.095 | 4.036 | 18.750 | 31.607 |
| 2021 | 2.036 | 53.370 | 4.060 | 23.560 | 41.369 |
| 2022 | 2.821 | 50.750 | 9.619 | 38.619 | 55.881 |
| 2023 | 4.976 | 46.643 | 14.238 | 61.155 | 66.821 |
| 2024 | 9.345 | 41.000 | 14.881 | 82.667 | 70.726 |
| 2025 | 4.250 | 40.381 | 5.155 | 82.833 | 74.488 |
| 2026 | 16.643 | 39.071 | 5.071 | 74.071 | 66.286 |

*Note: RSV = relative search volume derived from Google Trends. Values represent annual mean monthly RSV estimates for each search term in the United States. All values were rounded to three decimal places.
