# Supplementary material for: Temporal and seasonal trends in public interest in nonalcoholic beverages and lifestyles: A Google Trends analysis, 2004–2026
Source: PLoS One. 2026 Oct 29;21(10):e0361032. doi: 10.1371/journal.pone.0361032 (PMC13718958; doi:10.1371/journal.pone.0361032)
Supplement: Supplementary file 4 [file pone.0361032.s004.docx]

S4 Table. Seasonal patterns in monthly RSV across search terms

| Search term | Seasonal test F(df) | p value | Peak month | Peak adjusted RSV (95% CI) |
| --- | --- | --- | --- | --- |
| quit drinking | F (11, 252) = 8.70 | <0.001 | January | 47.789 (43.278, 52.299) |
| non alcoholic | F (11, 252) = 10.52 | <0.001 | December | 35.834 (32.791, 38.877) |
| mocktail | F (11, 252) = 1.26 | 0. 251 | N/A | N/A |
| sober curious | F (11, 252) = 0.97 | 0. 478 | N/A | N/A |
| dry January | F (11, 252) = 0.95 | 0. 498 | N/A | N/A |

*Note. Seasonal patterns were assessed using Newey–West regression models with calendar-month indicators and a 12-month lag. Models adjusted for long-term temporal trends using linear and quadratic terms for continuous time measured in years from January 2004. Overall seasonality was evaluated using joint *F* tests of the calendar-month indicators. Adjusted monthly RSV estimates were obtained using predictive margins, and peak months are reported only for statistically significant seasonal patterns. RSV = relative search volume; CI = confidence interval.
