# Supplementary material for: Temporal and seasonal trends in public interest in nonalcoholic beverages and lifestyles: A Google Trends analysis, 2004–2026
Source: PLoS One. 2026 Oct 29;21(10):e0361032. doi: 10.1371/journal.pone.0361032 (PMC13718958; doi:10.1371/journal.pone.0361032)
Supplement: Supplementary file 5 [file pone.0361032.s005.docx]

S5 Fig. Comparison of primary quadratic and restricted cubic spline models for long-term temporal trends

**Note.* Figures compare the long-term temporal patterns estimated using the primary quadratic models and restricted cubic spline sensitivity models for each search term. All models were estimated using monthly observations from January 2004 through February 2026 (*N* = 266), with time modeled continuously in years from January 2004 and monthly observations represented as fractional years. Models included calendar-month indicators to account for seasonality and used Newey–West standard errors with a 12-month lag. Trend lines display the seasonality-adjusted temporal component. The restricted cubic spline models used four knots placed at the 5th, 35th, 65th, and 95th percentiles of the observed time distribution.
