# Supplementary material for: Temporal and seasonal trends in public interest in nonalcoholic beverages and lifestyles: A Google Trends analysis, 2004–2026
Source: PLoS One. 2026 Oct 29;21(10):e0361032. doi: 10.1371/journal.pone.0361032 (PMC13718958; doi:10.1371/journal.pone.0361032)
Supplement: Supplementary file 6 [file pone.0361032.s006.docx]

S6 Fig. Long-Term Temporal Trends Estimated Using Restricted Cubic Spline and COVID-19 Segmented Regression Models

**Note.* The upper panel presents seasonality-adjusted temporal trends estimated using restricted cubic spline models with four knots placed at the 5th, 35th, 65th, and 95th percentiles of the observed time distribution. The lower panel presents trends estimated using segmented regression models with March 2020 specified as the pandemic-related breakpoint; the vertical dashed line indicates the breakpoint. Solid and dashed lines represent estimated pre- and post-breakpoint trends, respectively. All models were estimated using monthly observations from January 2004 through February 2026, included calendar-month indicators to account for seasonality, and used Newey–West heteroskedasticity- and autocorrelation-consistent standard errors with a 12-month lag. RSV = relative search volume.
